# Effects of resveratrol on reducing spermatogenic dysfunction caused by high-intensity exercise

**DOI:** 10.1186/s12958-019-0486-7

**Published:** 2019-05-06

**Authors:** Yuping Guo, Anli Wang, Xinpeng Liu, Enzhong Li

**Affiliations:** 1 School of Physical Education, Anyang Normal University, 436 Xiange Road, Anyang, 455000 Henan China; 2 School of Biological and Food Processing Engineering, Huanghuai University, 76 Kuaiyuan Road, Zhumadian, 463000 Henan China; 3 Beijing Sport University, 18 Xinxi Road, Beijing, China

**Keywords:** Resveratrol, High-intensity exercise, Spermatogenesis, iTRAQ proteomic analysis

## Abstract

**Background:**

Long-term high-intensity exercise can lead to reproductive endocrine and spermatogenic dysfunction. This research is to investigate the effect of resveratrol on the reduction of reproductive dysfunction induced by high-intensity exercise, and to screen relevant factors and signal transduction pathways.

**Methods:**

Rats were randomly divided into three groups, a control group, an intensive exercise group (IE group), and a resveratrol-treated group (RSV group). After 9 weeks of exercise, the sperm density and reproductive hormone concentrations were measured, along with antioxidation, inflammatory cytokine production, and histological analyses performed for each group. In addition, a proteomics analysis of the IE group and RSV group were conducted.

**Results:**

We found that compared with the control group, the average sperm density (*P* < 0.05) and testosterone concentration (P < 0.05) in the IE group decreased significantly. Additionally, in testis tissue the concentration of the inflammatory cytokines IL-6 (*P* < 0.01) and TNF-α (P < 0.01) increased significantly. Also, a significant decrease in superoxide dismutase (SOD) activity (*P* < 0.01) and a significant increase in the malondialdehyde (MDA) concentration (*P* < 0.01) were noted. In the RSV group, the average sperm density (*P* < 0.01), testosterone (P < 0.01) and follicle stimulating hormone (FSH) levels (*P* < 0.01) all increased in comparison to the IE group, and the concentration of IL-6 (*P* < 0.01) and TNF-α (P < 0.01) were found to be significantly decreased. Compared with the IE group, the SOD activity in the RSV group was significantly increased (P < 0.01), while the MDA content decreased (P < 0.01). Furthermore, histological analysis showed that the number of spermatogenic epithelial cells in the RSV group was higher than that of the IE group. There were a number of spermatogenic regulatory proteins identified in the proteomics analysis, including Clusterin, Piwi like homolog 1 (Piwil1), Zona pellucida binding protein (Zpbp), Heat shock-related 70 kDa protein 2 (Hspa2), Centrin 1, and Bardet-Biedl syndrome 2 protein (Bbs2). It was found that the proteins that differed between the two groups were mainly involved in pathways such as complement and coagulation cascades, the extracellular matrix-receptor interactions, etc.

**Conclusions:**

The present study demonstrates that after high-intensity exercise, the inflammatory cascade in the tissue of the testis increases with decreased resistance to oxidation and disordered spermatogenic function. Resveratrol can improve the reproductive dysfunction of rats that was induced by high-intensity exercise. It mostly promotes reproductive function by increasing testosterone secretion, reducing the inflammatory response, improving the antioxidant capacity, affecting the expression of spermatogenic regulatory proteins, and enhancing the signal transduction pathway of spermatogenesis.

## Background

In recent years, with an elevated level of competitive sports, the training load of athletes has increased accordingly. Moderate exercise not only improves cardiopulmonary function, but also enables adaptive changes such as an increase in the number of mitochondria in the muscle cells and capillaries in the muscles. However, long-term high-intensity exercise may cause health problems in athletes with either clinical or subclinical symptoms [1]. Previous studies have found that long-term high-intensity exercise training affects neuro-endocrine function, leading to a condition called “exercise-hypogonadal male condition”, as well as reduced spermatogenic function [2].

Although the exact physiological mechanism underlying reproductive dysfunction caused by the high volume exercise is currently unclear, a number of investigations have speculated that such reproductive dysfunction is associated with the hypothalamus and pituitary (central mechanism component) or the testes (peripheral mechanism component) of hypothalamic–pituitary–gonadal regulatory axis [3, 4]. In addition, intensive exercise causes increased production of reactive oxygen species. Then, oxidative stress may induced impairment in male reproductive system, which has a consequential effect upon testicular steroidogenesis and spermatogenesis [5].

Resveratrol (3, 4′, 5 trihydroxystilbene) is a natural non-flavonoid polyphenol compound, mainly found in plants such as grapes, veratrum and Japanese knotweed. Resveratrol is well absorbed, rapidly metabolized, and eliminated mainly through the urine. There are structures of the cis and trans in nature. Resveratrol has a variety of known benefits including anti-inflammatory properties, antioxidant, anti-tumor, cardiovascular protection, and promotion of reproductive function [6–10]. Resveratrol has been reported to exhibits anti-inflammatory activity through modulation of enzymes and pathways that produce mediators of inflammation [11]. Some studies have shown that Resveratrol inhibits tumorigenesis and metastasis of many cancers including mammary tumor, prostate and pancreatic cancers [7]. In addition, it has been demonstrated that Resveratrol obviously ameliorated spermatogonial stem cells (SSCs) loss to recover the spermatogenesis [12]. In recent years, the role of resveratrol in excercise training is gradually recognized. Studies have found that resveratrol has a positive effect on muscle strength, anti-fatigue ability, and the ability of endurance sports [13–15]. Previously, we found that low concentrations of resveratrol can restore the reproductive ability of cryptorchidism mice [10]. In this study, the effect of resveratrol on the high-intensity exercise induced decline of reproductive function was investigated, through both an inflammatory factor and antioxidant analysis. Furthermore, isobaric tag for relative and absolute quantitation (iTRAQ) proteomics was used to identify possible influencing factors and signal transduction pathways in this process, to explore the cause of the decline to spermatogenic function due to high-intensity exercise.

## Methods

### Ethical issues

The experimental protocol was approved by the Animal Experimental Committee of Huanghuai University, which were consistent with the guidelines provided by the Chinese National Guidelines for Animal Care.

### Animals

The experiment was performed using a total of 18 four-week-old male Sprague Dawley rats weighing (69.36 ± 5.13) g at the beginning of the experiment. The rats were randomly divided into a control group, an intensive exercise group (IE group), and a resveratrol treatment group (RSV group) (six rats per group). All rats were housed in a temperature-controlled room (25 ± 1 °C; 65% relative humidity and a 12: 12 light–dark cycle).

In the control group the rats were immersed in 32–35 °C water for 60 min per day for 9 weeks. Water depth reached the neck of the rats and they could move freely in the water. In the IE group, the rats were subjected to a swimming exercise with a weight of 6% of their body weight attached to the base of tail for 60 min per day. The exercise load was determined by reference to the literature and the exercise was performed 6 days per week for 9 weeks [16]. In the RSV group the animal exercise program was the same as the high-intensity exercise group. However, the rats were additionally treated with resveratrol every day until the end of the program. Animals were swimming in a swimming bucket (water depth 60 cm).

The RSV group was treated daily with 50 mg/kg/day resveratrol, purchased from Shaanxi Undersun Biomedtech, China (98% purity). Resveratrol was suspended in 0.2 ml of normal saline and given by gavage. Rats in the control and IE group were given the same amount of normal saline using the same method.

**Fig. 1:**
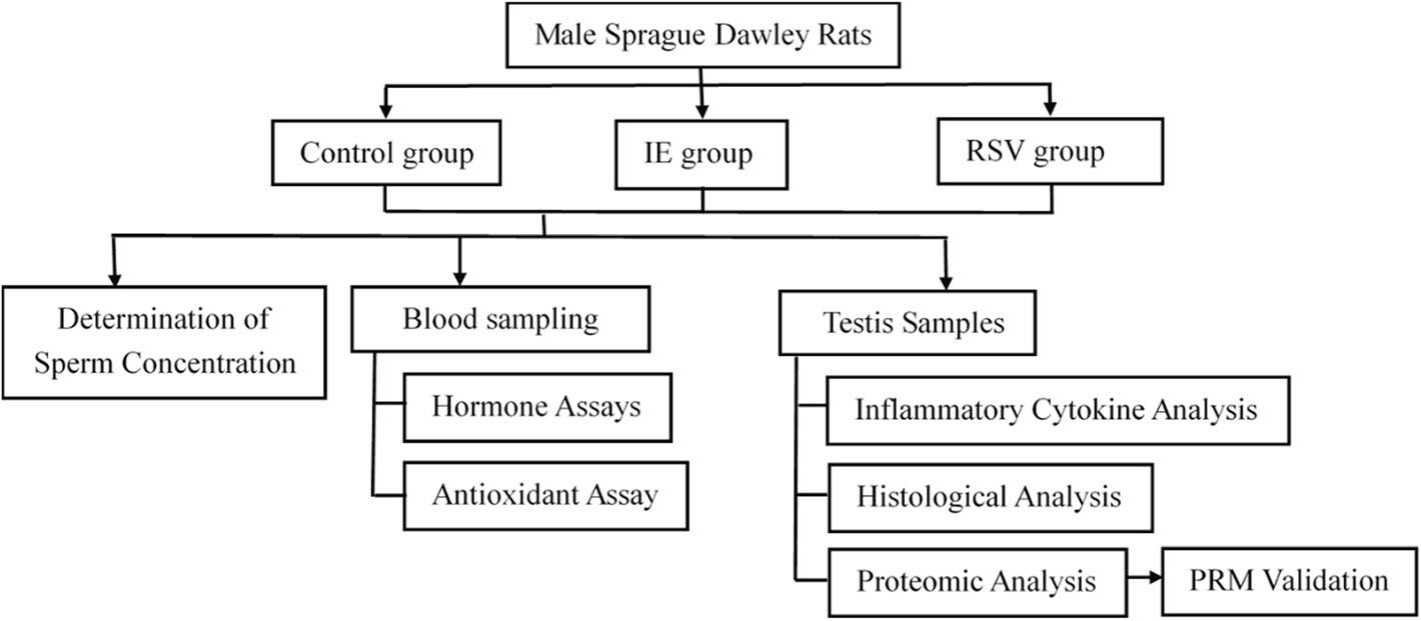
The experiment design

Figure 1 shows the experimental design.

### Hormone and antioxidant assay

Rats were anesthetized with ether and orbital blood was collected 24 h after the last exercise session. Blood samples were stored for 1 h at 4 °C before centrifugation for 10 min at 3500 g, and the serum supernatant was removed for hormone assays. Testosterone, gonadotropin releasing hormone (GnRH), luteinizing hormone (LH), and follicle stimulating hormone (FSH) test kits were purchased from Nanjing Jiancheng Bioengineering Co. Ltd. All hormone testing was carried out in accordance with the manufacturer’s guidelines. Measurement of Testosterone, GnRH, LH, and FSH levels were performed using the enzyme-linked immunosorbent assay (ELISA), the concentration of malondialdehyde (MDA) was measured using thiobarbituric acid colorimetry, and the superoxide dismutase (SOD) activity was determined using the xanthine oxidase method.

### Determination of sperm density

After the rats were killed by cervical dislocation, one side of the epididymis was carefully separated and placed in 2-ml of normal saline, cut into pieces. After being filtered, 2 ml normal saline at 32 °C was added, which is the optimum tempreture of rat epididymal sperm [17]. One drop of filtrate was taken and the sperm density was determined by the WLJY-9000 sperm quality detection system (Beijing Weili New Century Technology Development Co., Ltd.).

### Histological analysis

The left testicle of each rat was removed and fixed in Bouin’s solution for 12 h. Fixed samples were then rinsed three times with 50% ethanol (30 min per wash). Next, the testes were dehydrated in a gradient series of ethanol washes, treated with xylene, embedded in paraffin and sectioned. The testis samples were cut into 6 μm sections at 60 μm intervals. Sections were then stained with hematoxylin and eosin.

### Inflammatory cytokine analysis

The frozen testis samples were quickly homogenized in lysis buffer containing 50 mM Tris-Cl (pH 7.2), 150 mM NaCl, 0.1% SDS, 1% Triton X-100, 1 mM EDTA, 1 mM DTT, 1 μg/mL leupetin, 25 μg/mL aprotinin, and 1 mg/mL Phenylmethanesulfonyl fluoride (PMSF) at 4 °C for 20 min. The homogenate samples were then centrifuged at 13000 x *g* for 30 min at 4 °C. The supernatant was then taken to analyze the levels of interleukin-6 (IL-6) and tumor necrosis factor (TNF-α) in the testes tissues using ELISA kits. (Nanjing Jiancheng Bioengineering Institute).

### Proteomic analysis

After the rats were killed, the right testicles of the IE group and the RSV group were snap-frozen in liquid nitrogen and stored at − 80 °C to await proteomics analysis with iTRAQ technology.

Samples were first pulverized in liquid nitrogen. The dry powder was then dissolved in 200 μl of tetraethylammonium bromide (TEAB) dissolution buffer, and lysed by the ultrasonic waves for 15 min, and then centrifuged at 13,000 g for 20 min. Next, the supernatant precipitated by adding a 4-fold volume of cold acetone containing 10 mM dithiothreitol for 2 h. After centrifugation at 13,000 g for 20 min at 4 °C, the precipitate was collected, mixed with 800 μl of cold acetone and vortexed. Again, the sample was centrifuged at 13,000 g for 20 min at 4 °C and dried, the dried precipitate was collected and dissolved with 100 μl TEAB dissolution buffer and then stored at − 80 °C subsequent analysis.

Total protein concentration was measured using the Bradford method. For each pooled sample, 100 μg of protein was dissolved to 100 μl in a dissolution buffer, and then diluted with 500 μl 50 mM NH_4_HCO_3_. After samples were reduced and alkylated, 2 μg of trypsin was added and the samples were incubated overnight at 37 °C for protein digestion. After protein digestion, an equal volume of 0.1% formic acid (FA) was added for acidification. Peptides were purified on a Strata–X C18 pillar which was first activated with methanol and then balanced by adding 1 ml of 0.1% FA (three times), washed with 0.1% FA + 5% acetonitrile (ACN) twice, and finally eluted with 1 ml 0.1% FA + 80% ACN. Eluted peptides were dried with a vacuum concentration meter. The dried peptide powder was then re-dissolved with 20 μl of 0.5 M TEAB for peptide labeling.

Samples were labeled with an iTRAQ Reagent Multiplex Kit (AB SCIEX U.K. Limited) according to the manufacturer’s instructions. All of the labeled samples were mixed in equal amounts. Then, the peptide mixture was fractionated using a high-performance liquid chromatography system (Thermo DINOEX Ultimate 3000 BioRS) (Thermo Scientific Ltd.) using a Durashell C18 column (5 μm, 100 Å, 4.6 × 250 mm) under high pH conditions. The two mobile phases were buffer A, consisting of 20 mM ammonium formate in water (pH 10), and buffer B, consisting of 20 mM ammonium formate in ACN (pH 10). Each fraction was vacuum dried and stored at − 20 °C until MS analysis.

LC-ESI-MS/MS analysis was performed on a Triple Time-of-flight (TOF) 5600 plus AB SCIEX nanoLC-MS/MS system (AB SCIEX U.K. Limited). Samples underwent chromatography using a 90 min gradient from 2 to 30% (buffer A 0.1% [v/v] FA, 5% [v/v] ACN, buffer B 0.1% [v/v] FA, 95% [v/v] ACN) after direct injection onto a 20 μm PicoFrit emitter packed to 12 cm with Magic C18 AQ 3 μm 120 Å stationary phase. MS1 spectra were collected in the 350–1500 m/z range for 250 ms. The 20 most intense precursors with a charge state of 2–5 were selected for fragmentation, and MS2 spectra were collected in the 50–2000 m/z range for 100 ms; precursor ions were excluded from reselection for 15 s.

Bias correction and background correction were checked for protein quantification and normalization. An automatic decoy database search strategy was employed to estimate false discovery rate (FDR) using Proteomics System Performance Evaluation Pipeline software (AB SCIEX U.K. Limited), integrated in the ProteinPilot Software algorithm (AB SCIEX U.K. Limited). Protein identifications were accepted within the FDR of 1% in which at least one unique peptide match was specific for the protein. For protein abundance ratios measured using iTRAQ after normalization, we took a 1.5-fold change and a *P* value (Student’s t-test corrected for multiple testing Benjamini and Hochberg) < 0.05 as the threshold to identify significant changes. Analysis was performed using the protein database available at http://www.uniprot.org/. To determine the biological and functional properties of the identified proteins, protein sequences were mapped with Gene Ontology (GO) Terms (http://geneontology.org/); GO term matching was performed with blast2go v4.5 pipeline5. We used the hypergeometric test to perform GO enrichment and Kyoto Encyclopedia of Genes and Genomes (KEGG) pathway enrichment.

### Construction of the protein network interaction relationship

Differentially-expressed proteins were used to construct a Protein-Protein Interaction (PPI) network by searching the STRING database (http://www.string-db.org). The nodes and sides of the PPI represented different proteins and the protein-protein interactions, respectively. The degree (the number of sides connected to nodes) and betweenness (ratio of the shortest path through the nodes) in the PPI were calculated using CytoHubba.

### Parallel reaction monitoring MS

Based on the large-scale quantitative proteome study, proteins were selected for validation by targeted MS analysis using parallel reaction monitoring (PRM) on a Triple TOF 5600 + LC-MS/MS system (AB SCIEX U.K. Limited). Protein extraction and tryptic digestion were performed in the same way as for the iTRAQ experiment. ProteinPilot software was used to identify proteins, and the database search results were transferred into Skyline software for spectra library building. Target proteins for PRM validation were imported to Skyline software (Skyline Software Systems Inc.), and the peptides for protein quantification were selected according to ion signals in the spectra library. Data collection from each sample was performed using the final PRM acquisition method on the TOF mass spectrometer, where each precursor ion was selected by the quadrupole, fragmented in the collision cell, and then all fragment ions were quantified in the TOF mass analyzer. Data processing was carried out in Skyline, and the quantification results were manually inspected for each peptide of the targeted proteins. A fold change criterion (> 1.25) based on two times were reported as being significantly different.

### Statistical analysis

Quantitative data were expressed by mean ± standard deviation (M ± SD). Statistical analysis was performed using SPSS 19.0 statistical analysis software. Statistical analyses amongst the groups were conducted using a one-way ANOVA, and the comparison between any two groups was analysed using the Least-Significant Difference (LSD) t-test. A *P* < 0.05 was considered to represent a statistically significant difference, and a *P* < 0.01 indicated a very significant difference.

## Results

### Sperm count

It was found that there was a significant difference in sperm density amongst the groups. (P < 0.01). The results of the sperm density analysis showed that the average sperm density in the IE group [(1.47 ± 0.41) × 10^6^/ml] was significantly lower than that of the control group [(2.12 ± 0.43) × 10^6^/ml] (P < 0.05), and the sperm density in the RSV group [(2.75 ± 0.47) × 10^6^/ml] was significantly higher than that found in the IE group (P < 0.01).

### Histo-morphological analysis

**Fig. 2:**
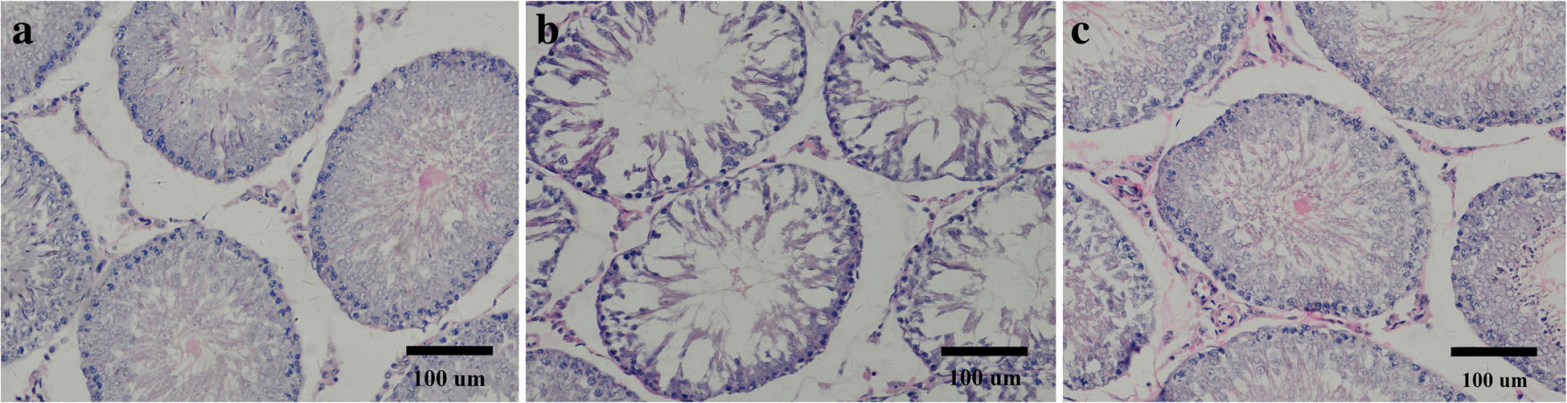
Representative images of histological sections of testes from rats in different group. **a** Control group; **b** IE group; **c** RSV group. The seminiferous tubules are full in the control and RSV group, but were slightly deformed in the IE group. The number of spermatogenic cells in the seminiferous tubules is lower in the IE group than in the Control and RSV groups

After hematoxylin and eosin (H&E) staining of testicular tissue isolated from each group, it was observed that the cells in each layer of the seminiferous tubules of the control group developed normally, with the layers also arranged in an orderly manner with large amounts of spermatozoa (Fig. 2). Whereas, the number of cell layers in samples taken from the IE group was found to be reduced when compared to the control group and RSV group. Additionally, the spermatogenic cells were loosely arranged, with a large number of vacuoles seen among the spermatogenic cells. However, the spermatogonia were more closely arranged in the RSV group compared to the IE group. Furthermore, the number of spermatogenic epithelial cells was observed to increase notably with a large number of spermatozoa.

### Hormone content determination

Analysis of the hormone concentration (Table 1) highlighted that there was a significant difference in the testosterone and FSH levels amongst the groups (*P* < 0.01), with the other hormones showing no significant difference (*P* > 0.05). It was found that after high-intensity exercise, the serum testosterone content in the IE group [(18.61 ± 1.02) nmol/l] was significantly reduced compared to the control group [(19.99 ± 0.25) nmol/l] (*P* < 0.05); however, there were no other significant changes to hormone concentrations (P > 0.05). The serum testosterone and FSH levels in the RSV group [(22.23 ± 0.82) nmol/l; (38.85 ± 1.32) mIU/ml] were significantly higher than that of the IE group [(18.61 ± 1.02) nmol/l; (35.43 ± 1.14) mIU/ml] (*P* < 0.01), but there was no significant change to the LH and GnRH concentrations (P > 0.05).

**Table 1:** Serum hormone concentrations in the Control, IE and RSV groups (*n* = 6)

Hormone	Testosterone (nmol/L)	FSH (mIU/ml)	LH (mIU/ml)	GnRH (ng/L)
Control group	19.99 ± 0.25	35.99 ± 1.34	18.84 ± 0.67	350.17 ± 36.22
IE group	18.61 ± 1.02^*^	35.43 ± 1.14	18.55 ± 0.66	348.50 ± 15.07
RSV group	22.23 ± 0.82^##^	38.85 ± 1.32^##^	19.18 ± 0.73	362.85 ± 20.58

Data are the mean ± s.d., ^*^P < 0.05 vs Control group, ^##^*P* < 0.01*vs* IE group

### Antioxidant test

As seen in Table 2, there was a significant difference in the SOD activity and MDA concentration amongst the groups (*P* < 0.01). Compared to the control group [(65.55 ± 3.87) active unit], the SOD activity in the IE group [(57.53 ± 6.38) active unit] was found to be significantly lower (*P* < 0.05); however, the plasma MDA concentration was found to be significantly increased [IE group: (5.74 ± 0.28) nmol/ml; control group: (4.70 ± 0.45) nmol/ml] (*P* < 0.01). The serum SOD activity in the RSV group [(86.69 ± 3.34) active unit] was noted to be significantly higher than that of IE group (*P* < 0.01), while the MDA content [RSV group: (3.01 ± 0.18) nmol/ml] was significantly decreased (P < 0.01).

**Table 2:** Results of antioxidant test (n = 6)

Group	Control group	IE group	RSV group
SOD (active unit)	65.55 ± 3.87	57.53 ± 6.38^*^	86.69 ± 3.34^##^
MDA (nmol / ml)	4.70 ± 0.45	5.74 ± 0.28^**^	3.01 ± 0.18^##^

* and ** indicate *P* < 0.05 and *P* < 0.01, respectively compared to the Control group; ^##^ indicates a *P* < 0.01 compared to the IE group

### Inflammatory cytokine analysis

**Fig. 3:**
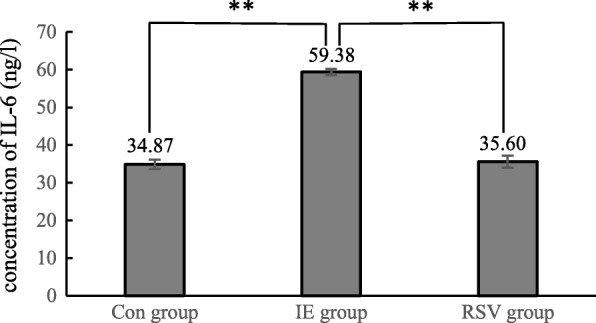
The level of IL-6 in the testes, * and ** indicates *P* < 0.05 and *P* < 0.01 compared with the control group

**Fig. 4:**
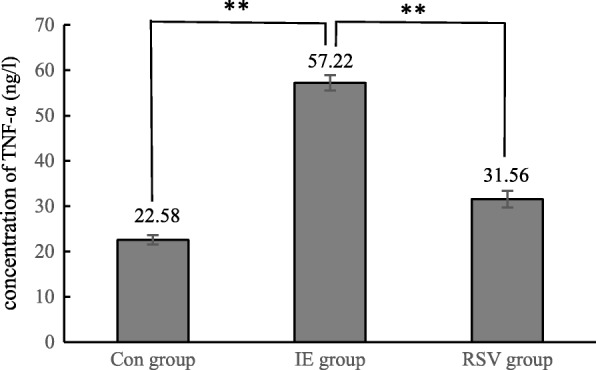
The level of TNF-α in the testes, * and ** indicates P < 0.05 and P < 0.01 compared with the control group

To study the inflammation after exercise, the levels of IL-6 and TNF-α in the testes of rats were measured. The results showed that the levels of IL-6 and TNF-α were significantly different amongst the groups (P < 0.01). The level of IL-6 and TNF-α in the IE group [(59.38 ± 0.83) ng/l; (57.22 ± 1.69) ng/l] was remarkably higher than that of the control group [(34.87 ± 1.26) ng/l; (22.58 ± 1.06) ng/l] (P < 0.01) and the RSV group [(35.60 ± 1.60) ng/l; (31.56 ± 1.83) ng/l] (P < 0.01). These results indicate that inflammation in the testicular tissue increased significantly after intensive exercise, and resveratrol had a therapeutic effect (Figs. 3 and 4).

### Statistical analysis of proteomics results

In order to explore the factors affecting reproductive dysfunction caused by high-intensity exercise, we compared proteomic differences between the RSV and IE group. The proteomic analysis revealed 236 differentially expressed proteins between the IE group and the RSV group. Among these, 139 differential proteins were up-regulated and 97 were down-regulated.

**Fig. 5:**
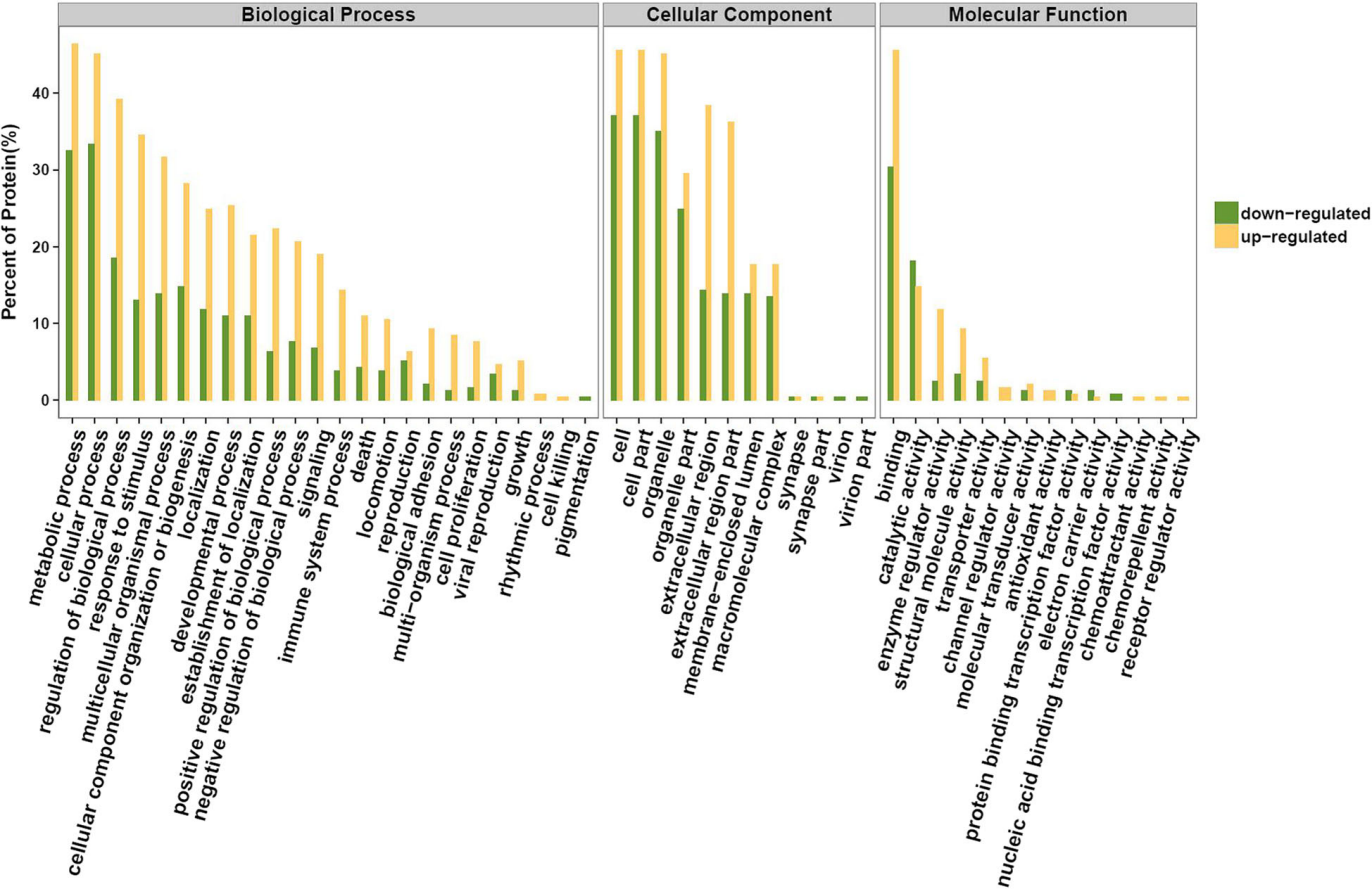
Comparison of upregulated and downregulated differentially expressed proteins according to Gene Ontology (GO) terms

The GO functional annotation was divided into three parts; biological process, molecular function, and cell components. As can be seen in Fig. 5, metabolic process was the primary biological process that the differentially expressed proteins were involved in, indicating that the addition of resveratrol affected basic tissue metabolism. It was also found in the differentially expressed proteins that a total of 15 were up-regulated and 12 were down-regulated in reproductive processes. Furthermore, 48 differentially expressed proteins were involved in immune system processes, of which 36 were up-regulated and 12 were down-regulated.

Enrichment analysis of differential proteins revealed that among the 27 differentially expressed proteins involved in the reproductive process 6 were enriched in biological processes involved in spermatogenesis, and these included Clusterin, Piwi like homolog 1 (Piwil1), Zona pellucida binding protein (Zpbp), Heat shock-related 70 kDa protein 2 (Hspa2), Centrin 1, and Bardet-Biedl syndrome 2 protein (Bbs2). Among the 48 differentially expressed proteins involved in immune system processes, 9 were found to be involved in the activation of the immune response, and these included the C9 protein, Complement C4, Complement C3, Complement component C8 beta chain, protein RGD1563231, LOC64038 protein, B-factor properdin, Ig lambda-2 chain C region, Protein Cfh, all of which were up-regulated.

**Fig. 6:**
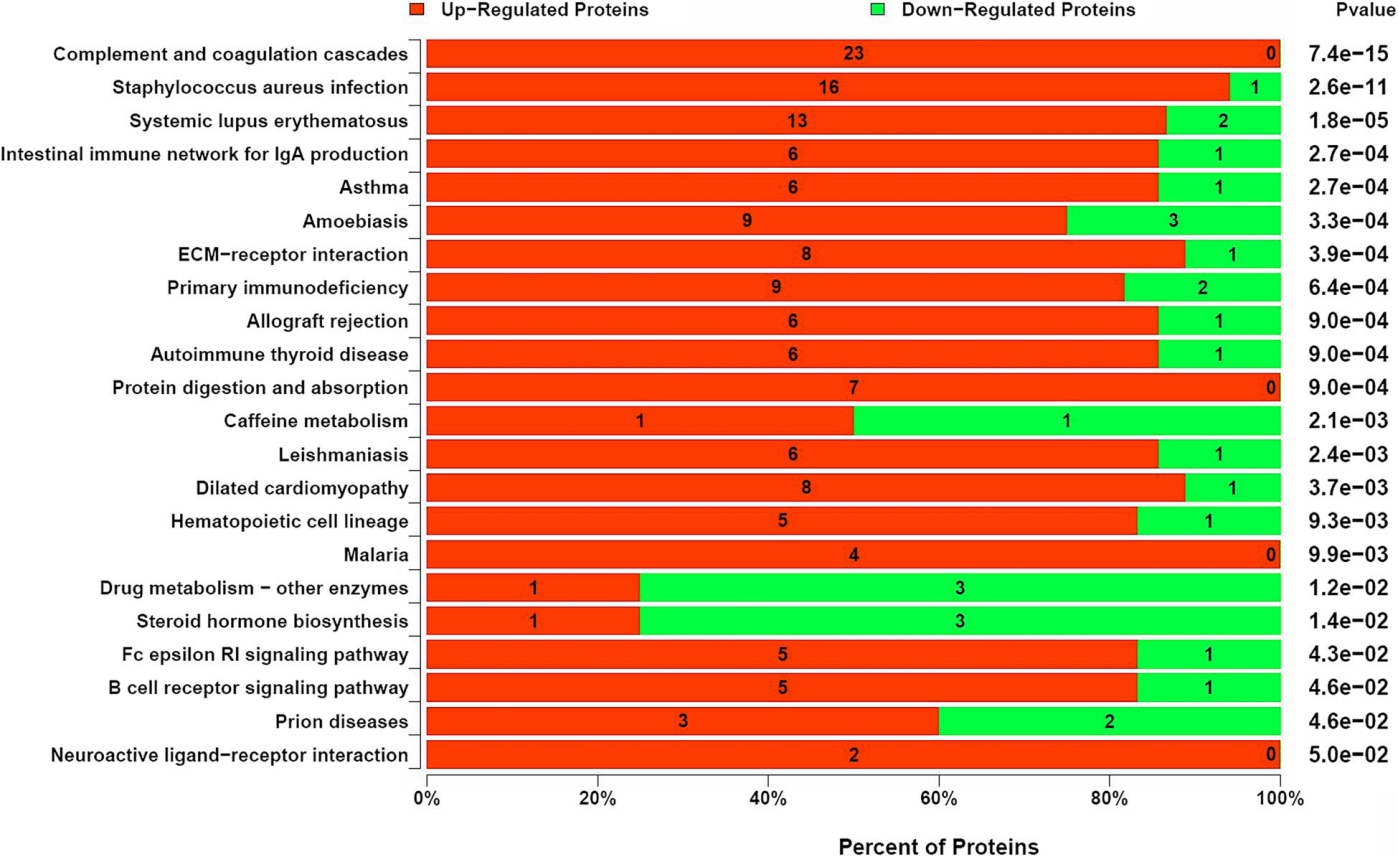
Results of pathway enrichment analysis highlighting the significance of differentially-expressed proteins. The numbers on the columns themselves represent the corresponding number of differentially expressed protein

**Fig. 7:**
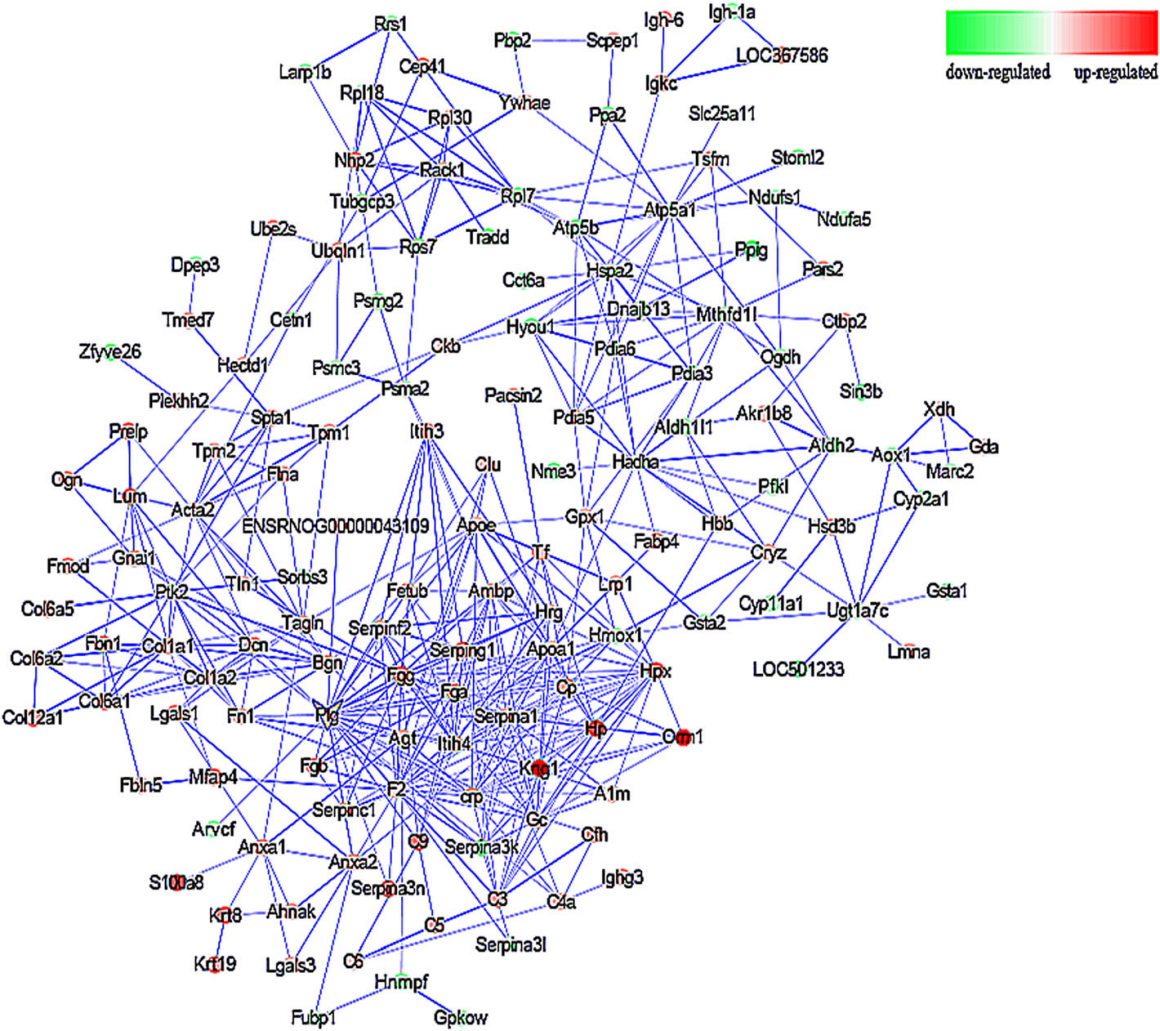
Differentially expressed gene network interaction diagram. The bigger the circle, the greater degree value of the gene in the network. The higher the confidence level of gene interaction, the thicker the connections between the nodes

Among the identified enriched pathways were the complement and coagulation pathway, *Staphylococcus aureus* infection, and systemic lupus erythematosus, primary immunodeficiency, Allograft rejection, B cell receptor signaling pathway, all of which are related to immune processes and inflammatory responses, and the extracellular matrix (ECM) receptor interaction pathway related to spermatogenesis (Fig. 6). In addition, PPI analysis was performed, as it can help elucidate the function of the identified protein in the entire tissue. An interaction network consisting of differentially expressed proteins was constructed and as can be seen in Fig. 7, one large network and six discrete protein connections were established. There are 168 points and 491 interactions formed in the network, with the central node identified as the gene that encodes for Plasminogen (Plg). The differentially expressed proteins, such as Haptoglobin, Collagen α-1(I) chain (Col1a1), Clusterin, Glutathione peroxidase 1 (Gpx1), and Complement C4 (C4a) found to be related to reproductive processes, antioxidant ability, and immunity were selected for parallel reaction monitoring (PRM) quantitative calibration and the results can be seen in Table 3. The PRM results were found to be consistent with the protein abundance changes quantified using iTRAQ.

**Table 3:** Targeted proteomics data validation by parallel reaction monitoring (PRM) and comparison with isobaric tags for relative and absolute quantification (iTRAQ) proteomics dat

Protein	PRM validation (fold-change)	iTRAQ proteomics data (Fold-change)
Haptoglobin	4.26	5.18
Col1a1	1.58	2.23
GPX1	1.36	1.54
Clusterin	0.75	0.64
C4a	2.41	1.89

## Discussion

High-intensity exercise promotes positive changes to functional movement, but it can also lead to adverse changes to certain tissues. Previous studies have shown that long-term high-intensity exercise can lead to neurological-endocrine disorders, resulting in exercise-induced low blood testosterone as well as decreased spermatogenesis and sperm quality [18–20].

Resveratrol is a polyphenol that is known to have a variety of biological effects. Previously, it has been shown that low concentrations of resveratrol can increase sperm production in cryptorchidism mice [8]. Therefore, the aim of this study was to explore the effect of resveratrol on high-intensity exercise induced spermatogenesis and its related mechanisms.

The results showed that after high-intensity exercise, compared to the control group, sperm concentration and testosterone concentration in the IE group decreased significantly, a large number of vacuoles appeared in the seminiferous tubules, and the number of spermatogenic cells decreased; all indicating that there was a decline of spermatogenic function due to high-intensity exercise. Additionally, in the RSV group, compared to the IE group, the serum FSH and testosterone concentrations as well as the sperm density were significantly increased. Furthermore, the germ cells in the spermatogenic epithelium were closely arranged and the spermatogenic cells increased, indicating that resveratrol can significantly improve spermatogenic dysfunction caused by high-intensity exercise.

The results of the antioxidant analysis showed that both the serum SOD activity decreased and MDA concentration increased significantly after high-intensity exercise, highlighting that high-intensity exercise increased oxidative stress and lipid peroxidation. Furthermore, it was found that the surface of the testicular tissue membrane, which is rich in polyunsaturated fatty acids, is more susceptible to oxidative stress damage [21]. Oxidative stress is known to destroy cell membrane structures and cause apoptosis [22]. The activity of SOD in the resveratrol treatment group was significantly improved, and the concentration of MDA decreased, indicating that resveratrol had a positive effect on the antioxidant capacity of the cells. Therefore, it is can be suggested that the cavity formed by the spermatogenic cells in the IE group may be due to an increase of oxidative stress after high-intensity exercise, leading to apoptosis of spermatogenic cells. Therefore, resveratrol treatment has the ability to enhance antioxidant capacity, and effectively improve any genital damage caused by oxidative stress during high-intensity exercise. However, the anti-oxidative stress mechanism of resveratrol is still not fully understood. Some studies have suggested that it may block the oxidative stress response by activating histone deacetylase and cause cellular transcriptional expression through the SIRT1/AMPK pathway [23, 24]; the specific mechanism of which requires further study.

According to the inflammatory cytokine analysis, inflammation in the testis was significantly enhanced in the IE group compared to the control group. This reinforces previous results that high-intensity exercise can cause inflammation [25]. Moreover, several studies have shown that systemic inflammation can inhibit testosterone secretion without relying on hypothalamic-pituitary levels, and also cause damage to the sperm [26, 27]. Therefore, it indicated that high-intensity exercise causes an increase in the inflammatory response in the testis tissue, and therefore causes the secretion of testosterone and spermatogenesis to be inhibited. Importantly, the inflammatory response was significantly decreased in the RSV group. Proteomic analysis showed that the expression of complement proteins such as Complement C4 and Complement C3 in the RSV group was up-regulated. In addition, the pathway enrichment analysis found that signaling pathways such as complement and coagulation cascades and *Staphylococcus aureus* infection, which are all associated with inflammation and immune responses, were mostly affected in this study. The results suggested that resveratrol after high-intensity exercise activates the immune response and reduces the inflammation caused by high-intensity exercise. Additionally, resveratrol increases the secretion of testosterone and FSH, and promotes germ cell differentiation and spermatogenesis through the up-regulation of complement expression. Testosterone and FSH are important hormones that maintain spermatogenesis, they play an important role in germ cell differentiation and sperm formation [28, 29].

In this study, the GO function enrichment analysis highlighted that there were differentially expressed proteins directly involved in spermatogenesis, including clusterin, Piwil1, Zpbp, Hspa2, Centrin 1 and Bbs2. Among the differentially expressed proteins, Collagen alpha-1(XII) chain and Collagen type VI alpha 1 chain, which are extracellular matrix components, were found to be both up-regulated. Furthermore, pathway significance enrichment analysis showed that the ECM-receptor interaction pathway is closely related to reproduction and is one of the more significant conduction pathways. Previous studies have shown that the ECM-receptor interaction pathway triggers signal transduction for processes such as differentiation and cell growth, through the interaction between the extracellular matrix and specific receptors on the cell surface [30]. Therefore, it can be speculated that the ECM-receptor interaction pathway is an important signal transduction pathway for improving high-intensity exercise induced reduction of spermatogenesis after the addition of resveratrol.

The central node gene of the differentially expressed protein interaction network in this study was the gene that encodes for the protein Plasminogen (Plg). Enrichment analysis of the biological processes of the *plg* gene showed that its main biological function was involved in the regulation of cell proliferation and cell adhesion; functions closely related to spermatogenesis. Furthermore, the known genes involved in spermatogenesis and reproductive regulation, including angiotensinogen and group specific components, form the central network in combination of *plg*, and were found to all be up-regulated in this study. Therefore, it is possible that these genes may regulate reproduction, spermatogenesis, and cell proliferation through a network of interactions in which plg plays a central role.

Based on a bioinformatics analysis and PPI analysis of differentially expressed proteins, we screened four differentially expressed proteins are related to spermatogenesis and reproductive function, which included Haptoglobin, Col1a1, Gpx1, and Clusterin. This was to investigate the possible differences in spermatogenic regulatory proteins after resveratrol treatment; however their specific functions require further studies.

Gpx1 is an important antioxidant enzyme, that not only scavenges reactive oxygen species but also repairs oxidized damaged cells. Additionally, it may also regulate cell differentiation and proliferation by regulating redox-sensitive signaling [31]. Previously it has been shown to have reduced expression in cancer cells [32, 33], highlighting a potential function in the regulation of cell division and differentiation. However, its role in the spermatogenesis process needs further exploration. Col1a1 is a component of the extracellular matrix that regulates the balance of self-renewal and differentiation of spermatogonial stem cells, playing an important role in spermatogenesis [34]. Therefore, it is speculated that the upregulation of Col1a1 can promote spermatogenesis. Haptoglobin is widely found in mammalian body fluids. Studies have shown that Haptoglobin is a mesenchymal cell and sperm cell product, which plays an important role in testicular ion metabolism [35]. Finally, clusterin is an important glycoprotein in semen, and to date two clusterin proteins, S1 and N1, have been identified. Studies investigating N1 are very limited, whereas S1 is generally considered a marker of abnormal sperm [36]. Therefore, it is suggested that the down-regulation of clusterin with the addition of resveratrol, may reduce the occurrence of high-intensity exercise induced abnormal spermatozoa.

## Conclusions

Based on the results of this study we conclude that resveratrol enhances reproductive function in four different ways after high-intensity exercise, by (1) increasing testosterone secretion and reproductive activity by reducing inflammation, (2) reducing tissue damage caused by high-intensity exercise and improving antioxidant capacity, (3) up-regulating the expression of protamine, and (4) strengthening the spermatogenic signaling pathway regulating the growth and differentiation of spermatogenic cells.
